# Stoichiometry of multi-specific immune checkpoint RNA Abs for T cell activation and tumor inhibition using ultra-stable RNA nanoparticles

**DOI:** 10.1016/j.omtn.2021.03.007

**Published:** 2021-03-13

**Authors:** Dan Shu, Long Zhang, Xuefeng Bai, Jianhua Yu, Peixuan Guo

**Affiliations:** 1 Center for RNA Nanobiotechnology and Nanomedicine, The Ohio State University, Columbus, OH 43210, USA; 2 College of Pharmacy, The Ohio State University, Columbus, OH 43210, USA; 3 College of Medicine, The Ohio State University, Columbus, OH 43210, USA; 4 Dorothy M. Davis Heart and Lung Research Institute, The Ohio State University, Columbus, OH 43210, USA; 5 NCI Comprehensive Cancer Center, The Ohio State University, Columbus, OH 43210, USA; 6 Department of Pathology, College of Medicine and Comprehensive Cancer Center, The Ohio State University, Columbus, OH 43210, USA; 7 Department of Hematology & Hematopoietic Cell Transplantation, City of Hope National Medical Center and Beckman Research Institute, Duarte, CA 91010, USA

**Keywords:** RNA nanotechnology, 3WJ nano-scaffold, immunotherapy, multi-specific drugs, Ab-like RNA NPs, checkpoint

## Abstract

Immunotherapy has become a revolutionary subject in cancer therapy during the past few years. Immune checkpoint-targeting antibodies (Abs) could boost anticancer immune responses. However, certain protein-based immunotherapies revealed side effects and unfavorable biodistribution, so effective non-protein options with lower side effects are highly sought after. RNA’s ability to form various three-dimensional configurations allows for the creation of a variety of ligands to bind different cell receptors. The rubber-like properties of RNA nanoparticles (NPs) allow for swift lodging to cancer vasculature with little accumulation in vital organs, resulting in a favorable pharmacokinetic/pharmacodynamic (PK/PD) profile and safe pharmacological parameters. Multi-specific drugs are expected to be the fourth wave of biopharmaceutical innovation. Herein, we report the development of multi-specific Ab-like RNA NPs carrying multiple ligands for immunotherapy. The stoichiometries and stereo conformations of the checkpoint-activating RNA NPs were optimized for T cell activation. When compared to mono- and bi-specific RNA NPs, the tri-specific Ab-like RNA NPs bound to the trimeric T cell receptor with the highest efficiency, showed the optimal T cell activation, and promoted the strongest anti-tumor function of immune cells. Animal trials demonstrated that the tri-specific RNA NPs inhibited cancer growth. This Ab-like RNA NP platform represents an alternative to protein Abs for tumor immunotherapy.

## Graphical abstract

**Figure:**
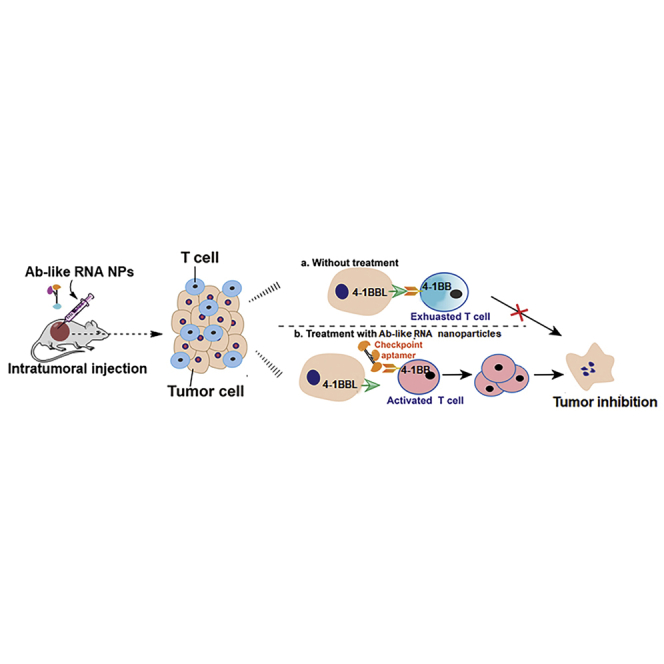

## Teaser

RNA therapeutics have become popular recently. Ab-like RNA nanoparticles could replace traditional protein antibodies in cancer immunotherapy and prove that matching the stoichiometry of the ligand RNA nanoparticles to the stoichiometry of the respective receptor subunits is very important in relationship to therapeutic efficiency.

## Introduction

Immunotherapy has become a popular point of research in the field of cancer treatment due to the discovery that monoclonal antibodies (mAbs) targeting co-inhibitory immune checkpoint proteins can improve the body’s immune response against cancer cells.^1^ Such checkpoint inhibitors restore T cell proliferation and stimulate effector functions, such as the release of effector cytokines and cytotoxic granules.^2^^,^^3^ Co-stimulatory checkpoint molecules, such as 4-1BB and CD28, are responsible for the proper activation of T lymphocytes.^4^ As of April 2018, about 25 agonist antibodies targeting immune co-stimulatory molecules have been clinically tested for use in cancer therapy.^4^

Evidently, the development of cancer immunotherapy was a major advancement in both the fields of immunology and oncology. However, some drawbacks limit the progress of the usual protein-based options in clinical trials. For example, Fc receptors of immunoglobulin (Ig)G-like bi-specific antibodies may be immunogenic, and non-specific interactions between bi-specific Abs with white blood cells may possibly change their bio-distribution.^5^ Additionally, toxicological effects such as immune-related adverse events (irAEs) pose a major challenge for immunomodulation attempts.^6^, ^7^, ^8^, ^9^

The amphoteric ionization property^10^ and intrinsic hydropathic nature of proteins^11^ make them relatively bulky. This leads them to often form aggregates in vital organs, resulting in nonspecific binding to vital organs. Such adverse effects can lead to systemic activation of the immune system,^12^ possibly causing fulminant and even fatal toxicological effects to occur with protein Abs.^13^ The known drawbacks of protein Abs can hamper their otherwise great therapeutic potential.^14^^,^^15^ Therefore, effective non-protein cancer-specific immunotherapy drugs are desirable as they may not produce such adverse effects.

Recent developments in protein Ab research and peptide pharmaceutics have made it possible to develop multi-specific protein Ab platforms to address the toxicity of immunotherapy and increase antibody efficacy.^15^ Multi-specific drugs have been predicted to be the fourth wave of biopharmaceutical innovation.^16^ Multi-specific Abs are re-engineered protein-based reagents with two or more variable regions that can bind both immune cells and cancer cells more precisely and effectively. As a result, they have achieved great success, such as in the cases of B cell acute lymphoblastic leukemia.^17^^,^^18^

The emerging protein-free aptamers represent a promising platform for targeted immunotherapy.^19^ Compared to protein-based immunotherapy mAbs, chemically synthesized aptamers possess many advantages as targeting reagents, including low cost, faster SELEX selection, low immunogenicity, rapid tissue penetration, and long-term stability.^20^ All well-known checkpoint molecules were developed as blocking aptamers and showed comparable effects to those of Abs in mouse models.^21^^,^^22^ However, one of the biggest obstacles in the development of such an aptamer delivery system is its various and complex scaffold selection system. Thus, the creation of a stable and flexible scaffold that can effectively deliver different aptamers for cancer immunotherapy is essential.

The concept of RNA nanotechnology was proposed in 1998,^23^^,^^24^ and it has now developed into a mature field with high potential as a novel therapeutic platform.^23^, ^24^, ^25^ RNA nanotechnology is the bottom-up self-assembly of nanometer-scale structures, which can include scaffolds, ligands, therapeutics, and regulators, composed mainly of RNA. By utilizing RNA nanotechnology, we have constructed a variety of RNA nanoparticles (NPs) and demonstrated that RNA NPs harboring different functional modules retain their folding capabilities and independent functionalities. The rubber-like properties of RNA NPs allow for swift lodging to cancer vasculature with little accumulation in vital organs, resulting in a favorable pharmacokinetic/pharmacodynamic (PK/PD) profile and safe pharmacological parameters.^26^ RNA-based nanostructures have diverse functions, including gene expression, gene regulation, and the specific binding to different molecules. Furthermore, due to promising advancements in the field, RNA nanotechnology can also use immunotherapy and immunomodulation applications.^27^, ^28^, ^29^, ^30^

Using the ultra-stable 3WJ DNA core from the packaging RNA (pRNA) of the phi29 bacteriophage as a scaffold, we have constructed robust multimeric RNA NPs with various tumor-targeting and immune checkpoint aptamers. We have previously demonstrated that the 3WJ scaffold used to create RNA NPs containing the EGFR,^31^ HER2,^32^^,^^33^ PSMA,^34^ annexin A2,^35^ CD133,^36^ and folate^37^ aptamers is able to bind to and enter tumor cells specifically and target solid tumors in animals with little or no accumulation in vital organs. The concept of using Ab-like RNA NPs for immunotherapy was brought about by the discovery that both protein Abs and popularly used phi29 pRNA core motifs displayed as a Y-shaped three-way junction. The multiple valency and specificity afforded to these RNA NPs can allow their use as Ab-like RNA NPs.

In this study, we designed, assembled, and characterized multi-specific Ab-like RNA NPs carrying immune checkpoint modulators using ultra-stable pRNA-3WJ as a nano-scaffold. We reconfirm that the pRNA-3WJ can be utilized as a nano-scaffold and is indeed a promising platform for aptamer-based checkpoint immunotherapy applications. We investigated the binding of multi-specific Ab-like RNA NPs to T cells and tumors cells. Optimization of stoichiometries and stereo conformations of checkpoint activators in the 3WJ scaffold was placed under scrutiny. It was found that Ab-like RNA NPs containing the 4-1BB aptamer could effectively stimulate T cell activation and proliferation *in vitro* and inhibit cancer growth in animal trials. The results suggested that the ultra-stable Y-shaped pRNA-3WJ nano-scaffold would be a promising vehicle for Ab-like RNA NP construction for the purpose of harboring RNA aptamers for immune checkpoint binding in cancer immunotherapy.

## Results

### Design, self-assembly, and physicochemical characterization of multi-specific Ab-like RNA NPs carrying multiple checkpoint activators

**Figure 1:**
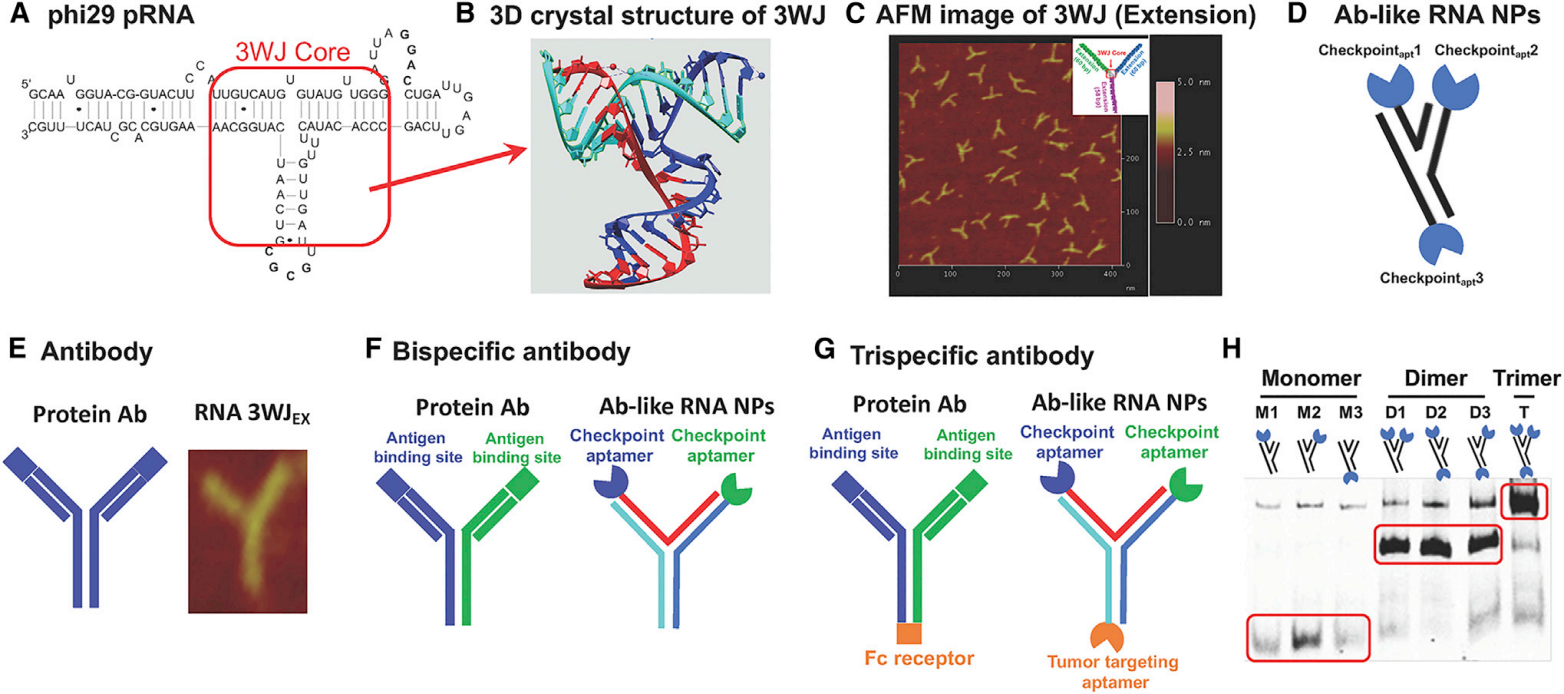
Comparison of protein Ab and Ab-like RNA NP platforms (A) pRNA-3WJ sequence. (B) Crystal structure showing the angles of pRNA-3WJ consisting of three ssRNA strands: red, blue, and cyan. (C) AFM image of 3WJ motif with extension. (D and E) Structure of protein Ab and Ab-like RNA NPs carrying checkpoint aptamers. (F and G) Comparison of bi-specific (F) and tri-specific (G) protein Ab and Ab-like RNA NP platforms. (H) Stepwise assembly of pRNA-3WJ with checkpoint RNA aptamers assayed by 8% native PAGE in TBM buffer (89mM Tris, 200mM Boric acid, 2.5mM MgCl_2_).

The concept of using Ab-like RNA NPs for immunotherapy was brought about by the discovery that both protein Abs and popularly used phi29 pRNA core motifs use a 3WJ scaffold (Figure 1).^38^ The nano-scaffold pRNA-3WJ core sequence was derived from the central domain of bacteriophage phi29 pRNA (Figure 1A). As shown in Figure 1B, this Y-shaped nano-scaffold can be assembled from three single-stranded RNA (ssRNA) oligonucleotides using a bottom-up self-assembly approach. Briefly, NPs were assembled by mixing the equal molar amounts of RNA fragments and gradually annealing from 85°C to 4°C in 1× RNA annealing buffer on a PCR machine. Atomic force microscopy (AFM) imaging clearly revealed the branched Y-shaped structure of the extended pRNA-3WJ (Figure 1C), which is similar to the shapes of protein Abs IgG, IgA, and IgE (Figure 1E). It was found that each arm of pRNA-3WJ could harbor one functional RNA module without affecting the folding of the central core and subsequent Y-shaped structure. This allows us to generate an RNA platform that consists of two or three different binding molecules fused into a single molecule similar to bi-specific and tri-specific protein antibody platforms (Figures 1F and 1G).

In order to develop a platform for immunotherapy using pRNA-3WJ as a nano-scaffold, immune checkpoint-targeting RNA aptamer sequences were incorporated into the 3WJ scaffold and annealed together to form Ab-like RNA NPs (Figure 1D). The 8% native PAGE gel electrophoresis showed a stepwise assembly of Ab-like RNA NPs from monomer (M) to dimer (D) and trimer (T) with high efficiency (Figure 1H).

### Binding of the multi-specific Ab-like RNA NPs to T cells and tumor cells *in vitro*

**Figure 2:**
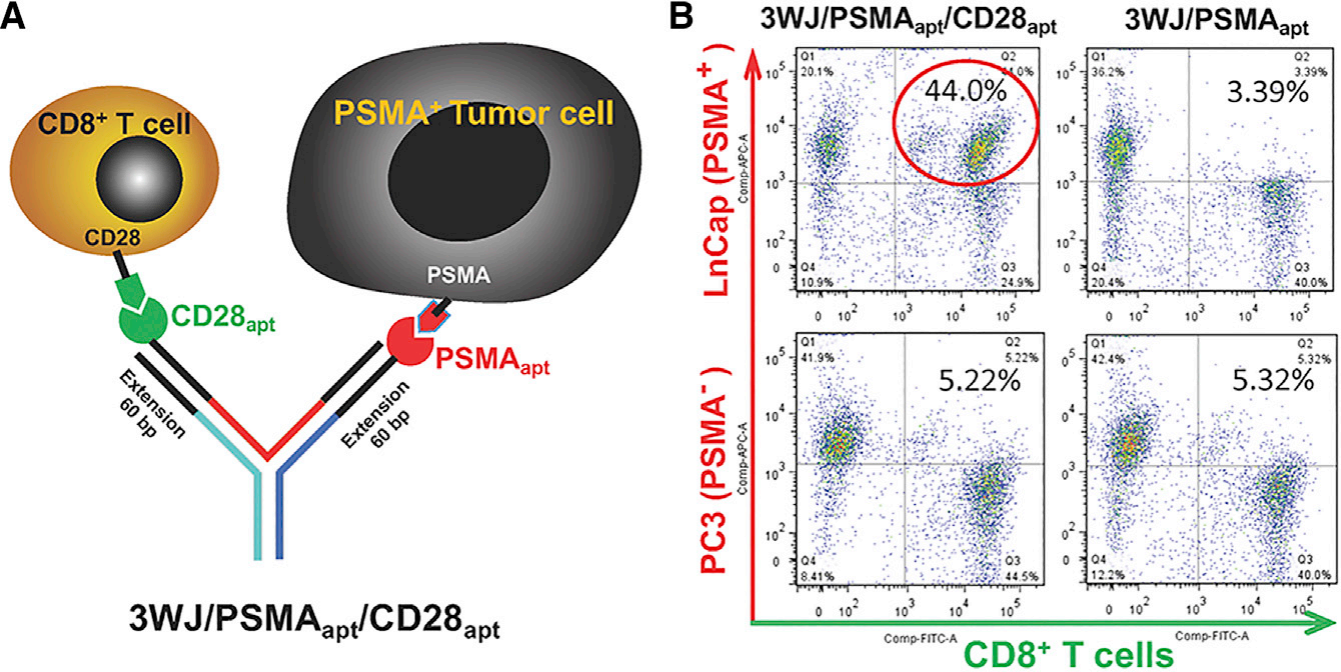
Analysis of bi-specific Ab-like RNA NP binding using flow cytometry (A) Schematic diagram of bi-specific Ab-like RNA NPs. (B) Flow cytometry results confirm the dual binding function of the bi-specific Ab-like RNA NPs to both T cells and tumor cells. Binding of bi-specific 3WJ/PSMA_apt_/CD28_apt_ to CD8^+^ T cells and LNCap tumor cells (PSMA^+^).

To explore the potential of using pRNA-3WJ nano-scaffolds for multi-specific Ab-like RNA NP immunotherapy, the extended 3WJ nano-scaffold was equipped with the anti-CD28 and anti-PSMA aptamers (Figure 2A). The selective bridging and binding capacities of 3WJ/PSMA_apt_/CD28_apt_ NPs to CD8^+^ T cells and PSMA^+^ tumor cells were determined using CellTracker Green CMFDA and Red CMTPX pre-stained CD8^+^ T cells and tumor cells, respectively. As shown in Figure 2B, the percentage of double cell tracker-positive cell populations in 3WJ/PSMA_apt_/CD28_apt_ RNA NP-treated LNCap (PSMA^+^) and CD8^+^ T cell mixtures was 44.0%, while it was 5.22% in PC3 (PSMA^−^) and CD8^+^ T cell mixtures. This result indicates that 3WJ/PSMA_apt_/CD28_apt_ RNA NPs could specifically bind to PSMA^+^ LNCap cells and CD8^+^ T cells and link them together. In contrast, the control RNA NPs without CD28 aptamer could not bridge CD8^+^ T cells and tumor cells in either PSMA^+^ LNCap or PSMA^−^ PC3 cells (Figure 2B).

### Comparison of the activity of mono-specific, bi-specific, and tri-specific Ab-like RNA NPs for the activation of T cells

**Figure 3:**
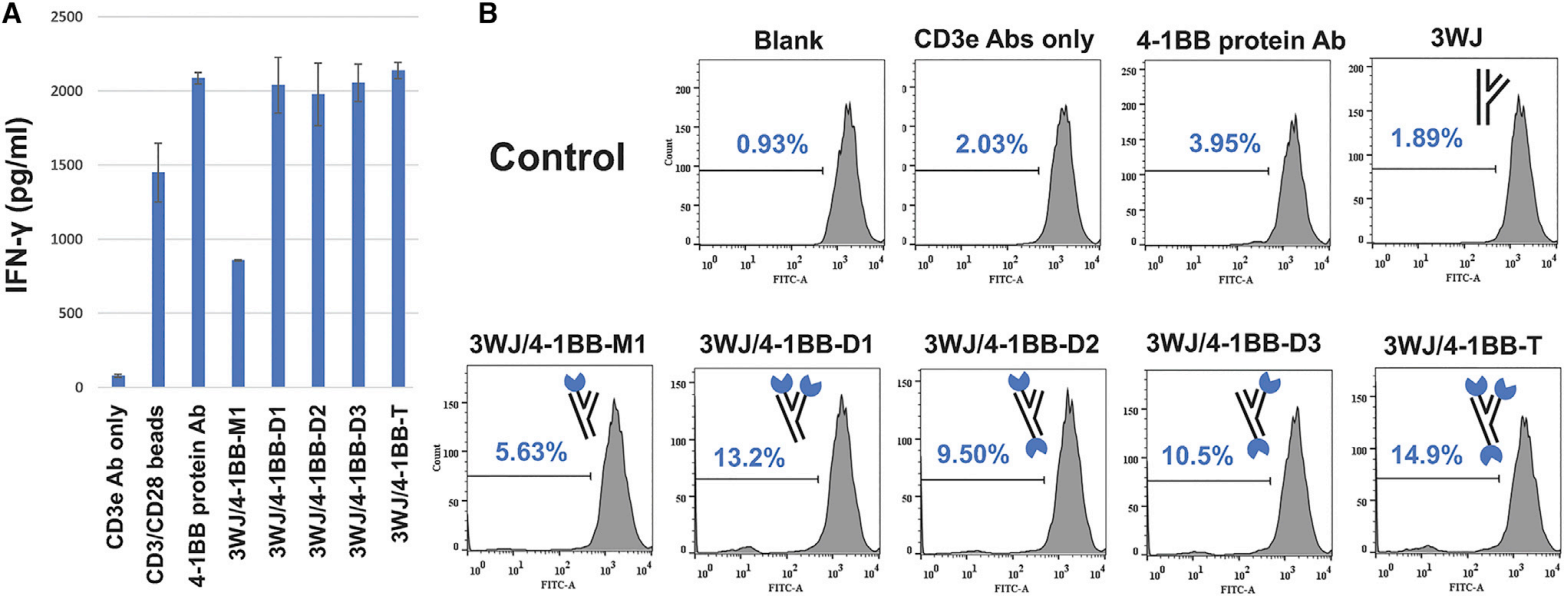
Analysis of CD8^+^ T cell activation and proliferation *in vitro* after 3WJ/4-1BB Ab-like RNA NP treatment (A) Evaluation of CD8^+^ T cells activation after 3WJ/4-1BB Ab-like RNA NP treatment via IFN-γ ELISA. (B) Evaluation of CD8^+^ T cell proliferation after 3WJ/4-1BB Ab-like RNA NP treatment via CFSE staining.

In order to determine whether different stoichiometries and stereo conformations of the 4-1BB aptamer in the 3WJ scaffold could deliver an enhanced co-stimulatory signal for the T cells, the 3WJ scaffold was designed in such a way as to carry different stoichiometric versions of the 4-1BB aptamer (monomer, dimer, and trimer), named as 3WJ/4-1BB-M, 3WJ/4-1BB-D, and 3WJ/4-1BB-T, respectively (Figure 1H). To test the immune activation capacity of different stereo conformations of the 4-1BB aptamer in the 3WJ scaffold, the 4-1BB dimer was placed in different arm locations of the 3WJ scaffold and named as 3WJ/4-1BB-D1, 3WJ/4-1BB-D2, and 3WJ/4-1BB-D3, respectively (Figure 1H). The proliferative capacity and secretion levels of interferon (IFN)-γ CD8^+^ T cells were measured after stimulation via different Ab-like RNA NP complexes. The secretion of IFN-γ from the activated CD8^+^ T cells was measured (Figure 3A). Incubation with the CD3/CD28 beads, 4-1BB protein Abs, or Ab-like RNA NPs, respectively, indeed induced IFN-γ secretion of CD8^+^ T cells.

The different stoichiometries of the 4-1BB aptamer in the 3WJ scaffold showed different IFN-γ induction capacities. The 3WJ/4-1BB-T Ab-like RNA NPs induced the highest level of IFN-γ in comparison to the Ab-like RNA NPs of 3WJ/4-1BB-D and 3WJ/4-1BB-M (Figure 3A). In contrast, 3WJ/4-1BB-D showed a more robust IFN-γ induction capacity than that of 3WJ/4-1BB-M. Notably, the level of IFN-γ in CD8^+^ T cells treated with 3WJ/4-1BB-T was higher than that of cells treated with 4-1BB protein Abs or CD3/CD28 beads (Figure 3A). The effect of different stereo conformations of the 4-1BB aptamer in the 3WJ scaffold on IFN-γ secretion was analyzed. The 4-1BB aptamer dimer was capable of co-stimulating T cell activation *in vitro* in different locations of the 3WJ scaffold with an efficiency comparable to that of the 4-1BB protein Abs (Figure 3A).

The effect of different stoichiometries and stereo conformations of the 4-1BB aptamer on CD8^+^ T cell proliferation was also studied. In this experiment, all Ab-like RNA NPs demonstrated better T cell activation than did 4-1BB protein Abs. 14.9% proliferation was observed in 3WJ/4-1BB-T RNA Ab-stimulated CD8^+^ T cells (Figure 3B). All three types of 3WJ/4-1BB-D Ab-like RNA NPs exhibited a better proliferative effect than that of 3WJ/4-1BB-M. In line with the results of IFN-γ induction, proliferation with 3WJ/4-1BB-D1, 3WJ/4-1BB-D3, and 3WJ/4-1BB-D2 reached 13.2%, 10.5%, and 9.5%, respectively, demonstrating the agonistic effects of different stereo conformations of the 4-1BB aptamer in the 3WJ scaffold on murine lymphocytes. No proliferation was observed without the anti-CD3 protein Ab stimulus, with the CD3e protein Abs alone, or with the CD3e protein Abs with just 3WJ (Figure 3B). These results indicate that the phi29 3WJ nano-scaffold is an ideal platform for the development of aptamer-based immunotherapy.

**Figure 4:**
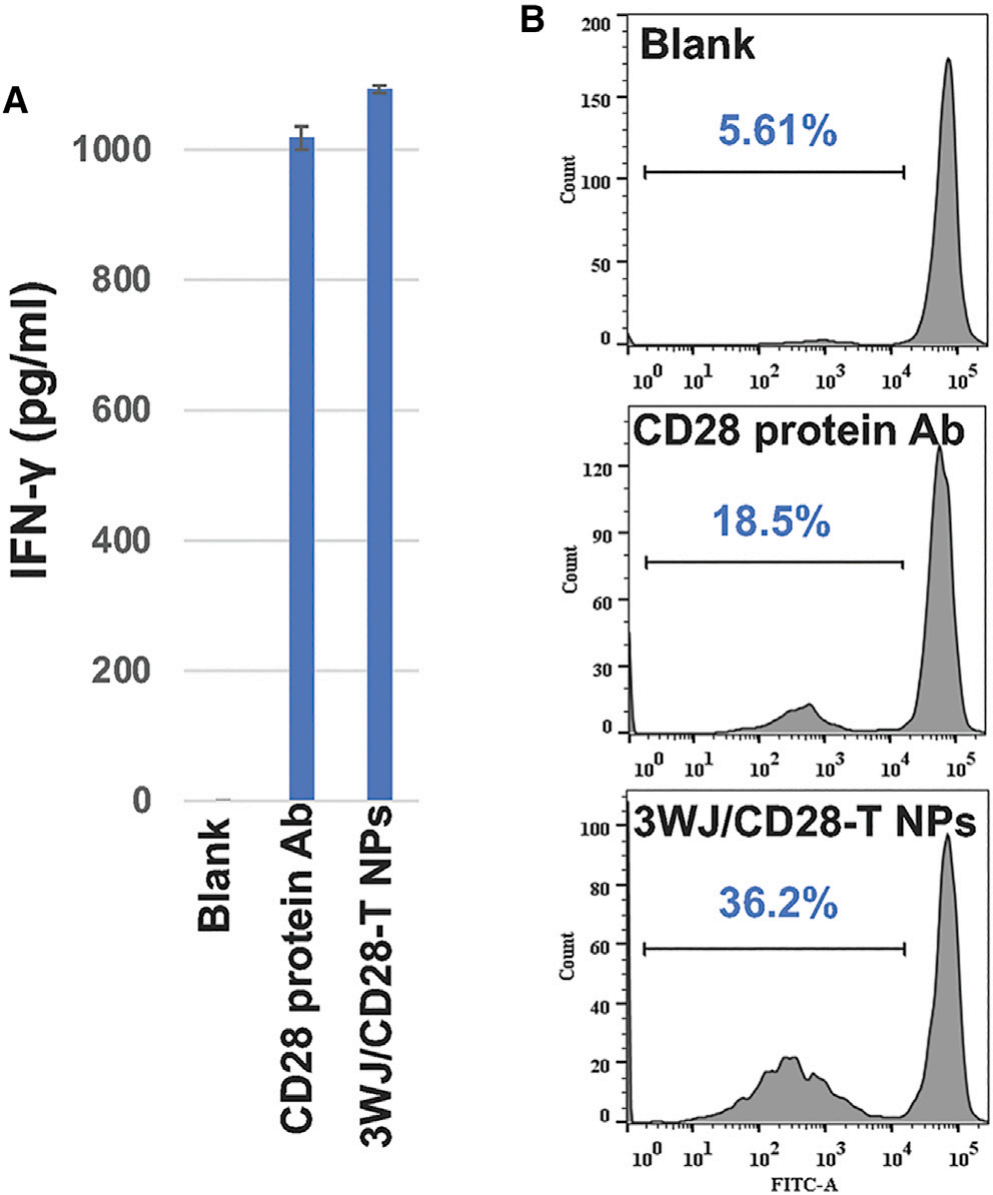
Analysis of CD8^+^ T cell activation and proliferation *in vitro* after 3WJ/CD28-T Ab-like RNA NP treatment (A) Evaluation of CD8^+^ T cell activation after 3WJ/CD28-T Ab-like RNA NP treatment via IFN-γ ELISA. (B) Evaluation of CD8^+^ T cell proliferation after 3WJ/CD28-T Ab-like RNA NP treatment via CFSE staining.

Most cell surface receptors assemble from three copies of protein subunits and display a trimeric configuration.^39^ To further investigate the stoichiometric properties of Ab-like RNA NPs and optimize the Ab-like RNA NPs characteristics for T cell activation, we constructed 3WJ/CD28-trimer_apt_ Ab-like RNA NPs (3WJ/CD28-T) and compared their IFN-γ induction and capacity for CD8^+^ T cell proliferation with that of CD28 protein mAbs. The 3WJ/CD28-T induced a more robust co-stimulatory signal than the CD28 protein mAbs (Figures 4A and 4B) as shown by a higher concentration of IFN-γ and percentage of cell proliferation. Additionally, proliferation with the 3WJ/CD28-T reached 36.2% of total cells, whereas the CD28 protein mAbs only reached 18.5% of the total cells, demonstrating the super-agonistic effects of anti-CD28 aptamers in 3WJ nano-scaffolds.

### Inhibition of cancer growth in animal trials via optimization of the stoichiometries and stereo conformations of the checkpoint activators in 3WJ nano-scaffolds

**Figure 5:**
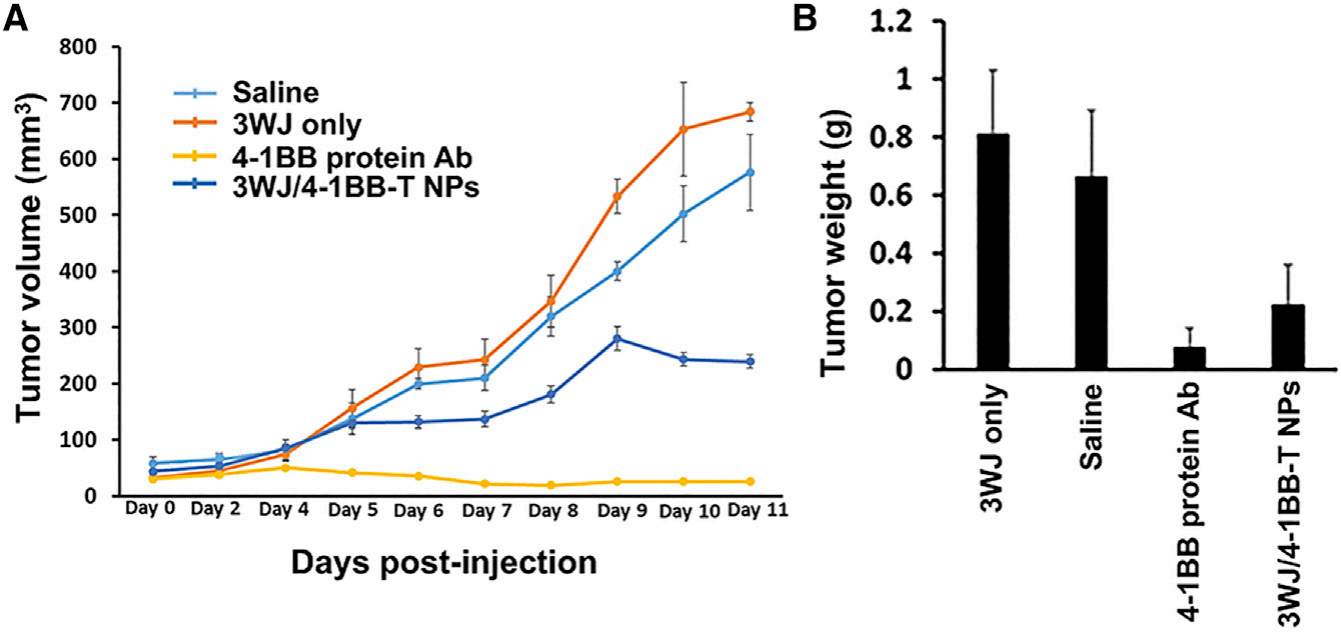
Evaluation of tumor inhibition effect *in vivo* (A) Tumor regression curve during the course of three injections. (B) Statistics of tumor weight after immunotherapy.

The *in vitro* results confirmed that the 4-1BB trimer was the most optimal stoichiometry and stereo conformation of the checkpoint activator in the 3WJ nano-scaffold. Next, we determined whether the 3WJ/4-1BB-T Ab-like RNA NPs were able to inhibit tumor growth in P815 mastocytoma-bearing mice via local intertumoral injection. Compared to the PBS and 3WJ-only groups, treatment with 3WJ/4-1BB-T Ab-like RNA NPs resulted in tumor inhibition from day 9 post-injection (Figure 5A). It is noteworthy that 4-1BB protein mAbs showed a better tumor inhibition effect *in vivo*. The tumor weight results also demonstrated the clear tumor inhibition effects of 3WJ/4-1BB-T Ab-like RNA NPs and 4-1BB protein mAbs (Figure 5B).

**Figure 6:**
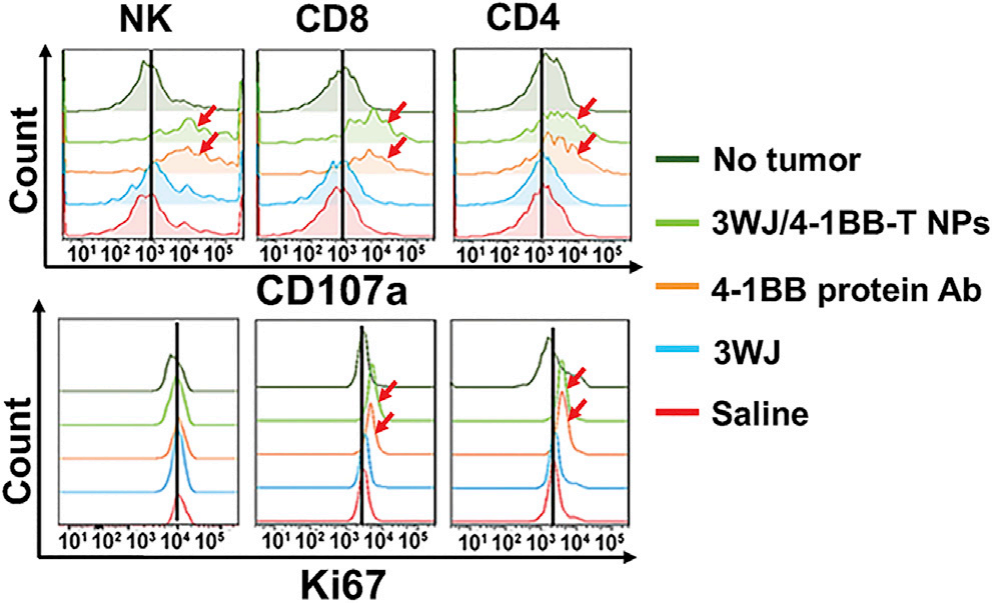
Evaluation of NK cell and T cell activation *in vivo* via flow cytometry

To explore the activation of the anti-tumor function of immune cells after Ab-like RNA NP treatment, we measured the ratio of degranulating T lymphocytes and natural killer (NK) cells in tumor via CD107a staining. The overlay histogram displayed an increased ratio of degranulating T lymphocytes and NK cells in the tumor of 3WJ/4-1BB-T Ab-like RNA NPs and 4-1BB protein mAb-treated mice (Figure 6), demonstrating the activation of the anti-tumor function of T lymphocytes and NK cells. The Ki67 staining results further served as proof of the activation of the immune cells, including T lymphocytes and NK cells (Figure 6). These results demonstrated that Ab-like RNA NP treatment could inhibit tumor growth via the enhancement of immune cell activation and the promotion of the degranulation of immune cells.

## Discussion

Cancer is a major public health problem worldwide and is the second leading cause of death in the United States. The burden of cancer in the United States is reflected by the fact 1,762,450 new cases and 606,880 related deaths were reported in 2019.^40^ Due to the increased understanding of immunology in the past few years, immunotherapies and checkpoint inhibitors have become popular subjects of research in the field of cancer treatment, revolutionizing the field of oncology. Co-stimulatory and co-inhibitory immune checkpoint molecules are important T cell modulators for anti-tumor immune responses. Co-inhibitory immune checkpoint molecules, such as PD-1, CTLA-4, TIM3, and LAG-3, expressed on T cells inhibit T cell activation and effector functions,^2^^,^^3^^,^^41^ while co-stimulatory checkpoint pathways, such as CD27, CD40, OX40, 4-1BB, GITR, ICOS, and CD28, are essential activators of T cell signaling pathways.^4^^,^^42^ Among the first-generation mAbs, the earliest CTLA-4 inhibitor ipilimumab and PD-1 inhibitor nivolumab/pembrolizumab have become first-line therapeutic options in advanced non-small cell lung cancer and melanoma cases.^43^^,^^44^

Engineered Abs hold great potential in cancer treatment. Nevertheless, as with other protein-based agents, their primary obstacle in terms of being effective immunotherapy options is their toxicity. For example, the first-line therapeutic ipilimumab is associated with autoimmune pathologies.^45^ Clinical trials using the highest dose of agonistic anti-4-1BB Abs in advanced cancer cases generated significant adverse effects, causing liver damage that resulted in several fatalities.^46^ To reduce side effects, immunotherapy drugs must specifically target the tumor or immune cells of the patient.^47^ Multi-specific drugs have been predicted to be the fourth wave of biopharmaceutical innovation, and four multi-specific drugs have been approved by the US Food and Drug Administration (FDA).^16^ Multi-specific Abs are one of the typical multi-specific drugs that can bind to multiple targets in order to eradicate tumor cells more precisely and effectively^5^. Due to rapid advancements in the biopharmaceutical industry, more than 120 multi-specific Abs are now in clinical use or going through clinical trials.^5^ Among them, catumaxomab, one of the first FDA-approved trifunctional Abs, binds CD3 on T cells, epithelial cell adhesion molecule (EpCAM) overexpressed on human adenocarcinomas, and the Fc receptors on other immune cells.^48^

However, the development of mAb-drug conjugates currently remains a challenge. Aptamer-based targeted therapy is an ideal supplemental platform to complement protein Abs for cancer immunotherapy. Indeed, several antagonistic aptamers are currently in clinical trials.^49^ Additionally, agonistic aptamers against 4-1BB, OX40, CD28, and CD40 were developed by the Gilboa laboratory with similar anti-tumor effects in mice to those of mAbs.^50^^,^^51^ Although both antagonistic and agonistic aptamers have shown comparable effects to those of Abs in mouse models, efficient delivery of antagonistic and agonistic aptamers on one modular scaffold remains a key challenge. For example, in the Gilboa laboratory, dimeric 4-1BB agonistic aptamers were constructed via the hybridization of two monomers with a 21-nt complementary linker. However, the same length link is invalid in the construction of dimeric CD28 agonistic aptamers, and OX40 agonistic aptamers require a different dimerization approach. The linker used in the tetravalent CTLA-4 aptamer construction is more complex.

In the present study, our results demonstrated that phi29-derived 3WJ pRNA is an ideal scaffold and platform for aptamer-based immunotherapy. We successfully constructed different Ab-like RNA NPs with various immune checkpoint-targeting aptamers. All of them were shown to be able to stimulate co-stimulatory signals, demonstrating comparable IFN-γ production and CD8^+^ T cell proliferation levels to those of commercial checkpoint protein mAbs. Exhilaratingly, both optimizations of the stoichiometries and stereo conformations of the 4-1BB and CD28 aptamers in the 3WJ nano-scaffold were shown to be better at stimulating CD8^+^ T cell proliferation capacity than protein mAbs *in vitro*. The main goal of this research is to test and determine which is better for RNA monomer, dimer, and trimer NPs in T cell activation. Previously published papers referenced in our article have shown that neither the mutant 4-1BB aptamers nor the scramble CD28 aptamers could bind to their target receptors or enhance T cell proliferation.^51^^,^^52^ Additionally, past publications also demonstrated that 3WJ scaffolds carrying scrambled aptamer sequences could neither bind to their receptors nor produce any treatment effects.^35^^,^^37^ So, in this study we did not design a 3WJ nano-scaffold with 4-1BB and CD28 mutant aptamers or scramble control. In this study, it was confirmed that 4-1BB and CD28 aptamer fused to the 3WJ scaffold can be well targeted to T cell surface receptors. Our recent study demonstrated the stretchable ability and rubbery property of RNA architectures, as proven using optical tweezers in stretching and release under external force and by the *in vivo* biodistribution assays. In contrast with DNA, RNA has higher elasticity and can fold into a wide range of shapes. However, it is necessary to consider that some aptamers need a specific distance to target the receptor. The structurally flexible RNA aptamer should target related receptors through self-adjustment of space when a flexible linker is added between the subunits. The length of the linker can be altered. The homotrimer 4-1BB ligand (4-1BBL) is a type II membrane protein and belongs to the tumor necrosis factor (TNF) superfamily.^53^^,^^54^ Three monomeric 4-1BBs (mono-4-1BBs) bind to a homotrimer 4-1BBL in the center,^39^ which results in increased expression of pro-survival molecules via nuclear factor κB (NF-κB) signaling.^55^ Both mono- and di-4-1BB are present on the T cell surface. The stoichiometry of the complex formed between di-4-1BB and tri-4-1BBL is a 2:1 mode of di-4-1BB to tri-4-1BBL.^39^ We speculated that 3WJ/4-1BB-T Ab-like RNA NPs could bind to both three mono-4-1BBs or two di-4-1BBs to enhance T cell activation. At the same time, 3WJ/4-1BB-D Ab-like RNA NPs can only bind to two di-4-1BBs. This means that 3WJ/4-1BB-T Ab-like RNA NPs can simultaneously stimulate the activation of the signal pathways of both mono- and di-4-1BB, while 3WJ/4-1BB-D Ab-like RNA NPs can only stimulate the activation of the di-4-1BB signaling pathway. This is the reason why 3WJ/4-1BB-T Ab-like RNA NPs possess a better T cell activation/proliferation capacity. Optimization of the stoichiometric and stereo conformational aspects of the 4-1BB aptamer resulted in tumor inhibition *in vivo* in a P815 mastocytoma-bearing mouse model. Moreover, past results indicate the potential for relatively safe administration of RNA NPs *in vivo*.^20^^,^^23^^,^^31^^,^^38^^,^^55^, ^56^, ^57^, ^58^

The ultra-stable pRNA-3WJ nano-scaffold system shows great potential for use in the development of immune checkpoint-based multi-specific RNA antibodies for cancer immunotherapy.

## Materials and methods

### Design, synthesis, self-assembly, and characterization of Ab-like RNA NPs carrying checkpoints and tumor-targeting aptamers

The short strands of the 3WJ core scaffold (3WJa, 3WJb, and 3WJc) were prepared via typical phosphoramidite oligonucleotide chemical synthesis using an automated oligonucleotide synthesizer. 2′-Fluorine-modified RNA strands with checkpoint or tumor-targeting aptamers were prepared as previously detailed using *in vitro* transcription with Y639F T7 polymerase. DNA strands used as primers and templates for double-stranded DNA (dsDNA) transcription templates were ordered from Integrated DNA Technologies (IDT) (Coralville, IA, USA).

**Table 1:** Sequence for Ab-like RNA NP construction

Name	Sequence (lowercase letters represent a 2′-fluorine modified base, underlined is 3WJ strand and italic is aptamer)
3WJ-a	5′-uuGccAuGuGuAuGuGGG-3′
3WJ-b	5′-cccAcAuAcuuuGuuGAucc-3′
3WJ-c	5′-GGAucAAucAuGGcAA-3′
3WJa (b or c)-4-1BB	5′-*GGGAGAGAGGAAGAGGGAuGGGcGAccGAAcGuGcccuucAAAGccGuucAcuAAccAGuGGc AuAAcccAGAGGucGAuAGuAcuGGAucccccc-*uuGccAuGuGuAuGuGGG-3' (3WJa) cccAcAuAcuuuGuuGAucc-3′(3WJb) GGAucAAucAuGGcAA-3′ (3WJc)
3WJa (b or c)-CD28	5′-*GGGAGAGAGGAAGAGGGAuGGGcAGAGAcuuccAAAAuAAAAGAcuccAuAAcccAGAGGucGAuAGuAcuGGAucccccc-*uuGccAuGuGuAuGuGGG-3′ (3WJa) cccAcAuAcuuuGuuGAucc-3′(3WJb) GGAucAAucAuGGcAA-3′ (3WJc)
Extended 3WJa-CD28_apt_	5′-*GGGAGAGAGGAAGAGGGAuGGGcAGAGAcuuccAAAAuAAAAGAcuccAuAAcccAGAGGucGAuAGuAcuGGAucccccc-*GGGAcAGcAcAcAGAGcAGcAGcuuGAGAcucAGcGuAcuucuGGcA AGGuAcGGuAcuuuuGccAuGuGuAuGuGGGcGcAGAcGGcGAuAccuAGuAGucAccuAGuGcucuAucGu AGAAGuGuAGcAuGAcGcc-3′
Extended 3WJb-PSMA_apt_	5′-*GGGAccGAAAAAGAccuGAcuucuAuAcuAAGucuAcGuuccc-*GGcGucAuGcuAcAcuucuAcGAu AGAGcAcuAGGuGAcuAcuAGGuAucGccGucuGcGcccAcAuAcuuuGuuGAucc-3′
Extended 3WJc	5′-GGAucAAucAuGGcAAAAGuAccGuAccuuGccAGAAGuAcGcuGAGucucAAGcuGcuGcucuGuG uGcuGuccc-3′

RNA transcripts were then purified using 8 M urea and 8% denaturing PAGE. All RNA NPs were self-assembled using a bottom-up approach and examined using 8% TBM-native PAGE as previously described. NPs with one, two or three aptamers were assembled by mixing three RNA strands with or without aptamer and annealing from 85°C to 4°C gradually in 1× RNA annealing buffer (10mM Tris, 50mM NaCl, 1mM EDTA, pH 7.5). The sequences are described in Table 1.

Multi-specific 3WJ/PSMA_apt_/CD28_apt_ NPs are composed of three strands with an extended 3WJ core scaffold (extended 3WJa-CD28_apt_, extended 3WJb-PSMA_apt_, and extended 3WJc). The sequences are described in Table 1.

### Separation and purification of spleen CD8^+^ T cell*s*

CD8^+^ T cells were separated and purified from the spleens of 4- to 6-week-old female BALB/c mice using an EasySep mouse CD8^+^ T cell isolation kit (STEMCELL Technologies, Cambridge, MA, USA). The single-cell suspension was prepared in modified Hanks’ balanced salt solution (HBSS) (Gibco, Carlsbad, CA, USA) containing 2% fetal bovine serum (FBS) (without Ca^2+^ and Mg^2+^) after lysis of the red blood cells (RBCs) in l× RBC lysis buffer. The purification was performed following the manufacturer’s instructions. At the end of the purification, CD8^+^ T cells were pelleted, resuspended in HBSS containing 2% FBS, and counted. The purified CD8^+^ T cells were plated in RPMI 1640 medium with 10% FBS for further study.

### Evaluation of T cell activation via enzyme-linked immunosorbent assay (ELISA)

Purified CD8^+^ T cells were seeded into 96-well U-bottomed plates (10^5^ cells/well) pre-coated with 5 μg/mL anti-CD3 (clone 145-2C11, eBioscience, San Diego, CA, USA). Anti-mouse CD28 (2 μg/mL) (clone 37.51, eBioscience), anti-mouse 4-1BB (5 μg/mL) (Leinco Technologies, St. Louis, MO, USA), and CD3/CD28 beads (Thermo Fisher Scientific, Waltham, MA, USA) were used as a positive control. 200 nM 3WJ only (control), 3WJ/4-1BB-M, 3WJ/4-1BB-D, 3WJ/4-1BB-T, and 3WJ/CD28-T were added to a final culture volume of 200 μL/well in complete RPMI 1640 medium and incubated at 37°C and 5% CO_2_ for 72 h. Afterward, 50 μL of cell culture supernatants was collected, and concentrations of IFN-γ were determined using a mouse IFN-γ DuoSet ELISA kit (R&D Systems, Minneapolis, MN, USA) following the protocol provided by the manufacturer. Data represent the mean ± standard deviation (SD) of three independent experiments.

### *In vitro* proliferation assays

The purified CD8^+^ T cells were labeled with 2 μM CFSE (5-(and 6)-carboxyfluorescein diacetate, succinimidyl ester; 1:1,000 dilution) using a CellTrace CFSE cell proliferation kit (Thermo Fisher Scientific) and incubated at 37°C for 20 min. After adding the complete culture medium, cells were incubated at 37°C for an additional 5 min. The cells were pelleted via centrifugation and plated immediately into 96-well U-bottomed plates (2 × 10^5^ cells/well) pre-coated with 5 μg/mL anti-CD3 (clone 145-2C11, eBioscience) in complete RPMI 1640 medium containing 10% FBS and IL-2 (50 U/mL). The cells were treated with an ELISA assay, as mentioned earlier, and after 3 days of culture, the cells were collected. *In vitro* proliferation was analyzed via fluorescence-activated cell sorting (FACS) analysis with a FACSCalibur flow cytometer. Data were analyzed using FlowJo.

### *In vitro* bi-specific binding assays

The linking and bridging capabilities of multi-specific 3WJ/PSMA_apt_/CD28_apt_ NPs were tested via FACS analysis using CellTracker Green CMFDA and Red CMTPX (Thermo Fisher Scientific). 3WJ/PSMA_apt_ NPs served as a control. Briefly, purified CD8^+^ T cells were labeled with CellTracker Green CMFDA (1:1,000 dilution), and PSMA-positive LNCap cells (or PSMA-negative PC3 cells) were labeled with CellTracker Red CMTPX (1:1,000 dilution). The CellTracker-labeled cells were incubated at 37°C for 30 min and then washed twice with PBS. Afterward, 10^5^ CD8^+^ T cells and tumor cells were incubated at a 1:1 ratio at 37°C in Opti-MEM containing 100 nM multi-specific 3WJ/PSMA_apt_/CD28_apt_ NPs for 2 h. The mixture system was then subjected to flow cytometry analysis using a FACSCalibur flow cytometer. The linking and bridging capabilities of 3WJ/PSMA_apt_/CD28_apt_ NPs were illustrated by comparing the percentage of double CellTracker-positive cell populations among the different groups. Data were analyzed using FlowJo.

### Animal study

All animal procedures were performed in accordance with the Subcommittee on Research Animal Care of The Ohio State University with guidelines approved by the Institutional Review Board. Female DBA/2 mice (4–6 weeks old) were purchased from Charles River Laboratories. P815 mastocytoma cells were resuspended in PBS at a concentration of 8 × 10^5^ cells/mL. 8 × 10^4^ cells were implanted subcutaneously into the flanks of mice. Mice were monitored every 48 h for tumor growth. When the length or width of tumors grew to average approximately 5 mm, the tumor-bearing mice were randomly divided into four groups (three mice/group) as follows: PBS control, 4-1BB protein Abs, 3WJ only, and 3WJ/4-1BB-T Ab-like RNA NPs. Mice were subsequently treated with 30 μg of total samples of 4-1BB protein Abs or Ab-like RNA NPs through intertumoral injections at a 2-day interval up to three times total. Tumor growth would then be monitored, and tumor size would be recorded with a digital caliper every day. At the end of the experiment, the mice were sacrificed, and tumors from terminated animals were collected and evaluated. The single-cell suspension was prepared in the tumor by homogenization. To evaluate immune cell antitumor activity, the single cells from tumor were stained with anti-CD3e, anti-CD4 or anti-CD8 or anti-CD56, and anti-CD107a to detect degranulating T lymphocytes and NK cells. To evaluate immune cell activation after 4-1BB protein Ab or 3WJ/4-1BB-T Ab-like RNA NP stimulation, the single cells were stained with anti-CD3e, anti-CD4 or anti-CD8 or anti-CD56, and anti-Ki67 Abs.
